# Deep learning based bilateral filtering for edge-preserving denoising of respiratory-gated PET

**DOI:** 10.1186/s40658-024-00661-z

**Published:** 2024-07-09

**Authors:** Jens Maus, Pavel Nikulin, Frank Hofheinz, Jan Petr, Anja Braune, Jörg Kotzerke, Jörg van den Hoff

**Affiliations:** 1 Department of Positron Emission Tomography, Institute of Radiopharmaceutical Cancer Research, Helmholtz-Zentrum Dresden-Rossendorf, Bautzner Landstraße 400, 01314 Dresden, Germany; 2 Klinik und Poliklinik für Nuklearmedizin, Universtitätsklinikum Carl Gustav Carus, Fetscherstraße 74, 01307 Dresden, Germany

**Keywords:** Positron emission tomography (PET), Image quantification, Deep learning, Post-filtering, Neural networks, Image denoising, Respiratory gating, Motion correction

## Abstract

**Background:**

Residual image noise is substantial in positron emission tomography (PET) and one of the factors limiting lesion detection, quantification, and overall image quality. Thus, improving noise reduction remains of considerable interest. This is especially true for respiratory-gated PET investigations. The only broadly used approach for noise reduction in PET imaging has been the application of low-pass filters, usually Gaussians, which however leads to loss of spatial resolution and increased partial volume effects affecting detectability of small lesions and quantitative data evaluation. The *bilateral filter* (BF) — a locally adaptive image filter — allows to reduce image noise while preserving well defined object edges but manual optimization of the filter parameters for a given PET scan can be tedious and time-consuming, hampering its clinical use. In this work we have investigated to what extent a suitable deep learning based approach can resolve this issue by training a suitable network with the target of reproducing the results of manually adjusted case-specific bilateral filtering.

**Methods:**

Altogether, 69 respiratory-gated clinical PET/CT scans with three different tracers ($[^{18}\text {F}]$FDG, $[^{18}\text {F}]$L-DOPA, $[^{68}\text {Ga}]$DOTATATE) were used for the present investigation. Prior to data processing, the gated data sets were split, resulting in a total of 552 single-gate image volumes. For each of these image volumes, four 3D ROIs were delineated: one ROI for image noise assessment and three ROIs for focal uptake (e.g. tumor lesions) measurements at different target/background contrast levels. An automated procedure was used to perform a brute force search of the two-dimensional BF parameter space for each data set to identify the “optimal” filter parameters to generate user-approved ground truth input data consisting of pairs of original and optimally BF filtered images. For reproducing the optimal BF filtering, we employed a modified 3D U-Net CNN incorporating *residual learning* principle. The network training and evaluation was performed using a 5-fold cross-validation scheme. The influence of filtering on lesion SUV quantification and image noise level was assessed by calculating absolute and fractional differences between the CNN, manual BF, or original (STD) data sets in the previously defined ROIs.

**Results:**

The automated procedure used for filter parameter determination chose adequate filter parameters for the majority of the data sets with only 19 patient data sets requiring manual tuning. Evaluation of the focal uptake ROIs revealed that CNN as well as BF based filtering essentially maintain the focal $\text {SUV}_\text {max}$ values of the unfiltered images with a low mean ± SD difference of $\delta \text {SUV}_\text {max}^{\text {CNN},\text {STD}}$ = (−3.9 ± 5.2)% and $\delta \text {SUV}_\text {max}^{\text {BF},\text {STD}}$ = (−4.4 ± 5.3)%. Regarding relative performance of CNN versus BF, both methods lead to very similar $\text {SUV}_\text {max}$ values in the vast majority of cases with an overall average difference of $\delta \text {SUV}_\text {max}^{\text {CNN},\text {BF}}$ = (0.5 ± 4.8)%. Evaluation of the noise properties showed that CNN filtering mostly satisfactorily reproduces the noise level and characteristics of BF with $\delta \text {Noise}^{\text {CNN},\text {BF}}$ = (5.6 ± 10.5)%. No significant tracer dependent differences between CNN and BF were observed.

**Conclusions:**

Our results show that a neural network based denoising can reproduce the results of a case by case optimized BF in a fully automated way. Apart from rare cases it led to images of practically identical quality regarding noise level, edge preservation, and signal recovery. We believe such a network might proof especially useful in the context of improved motion correction of respiratory-gated PET studies but could also help to establish BF-equivalent edge-preserving CNN filtering in clinical PET since it obviates time consuming manual BF parameter tuning.

**Supplementary Information:**

The online version contains supplementary material available at 10.1186/s40658-024-00661-z.

## Background

Even for state of the art systems, image noise in positron emission tomography (PET) remains inherently much higher than in computed tomography (CT) or magnetic resonance imaging (MRI), thus improving noise reduction techniques remains of considerable interest. This is especially true for respiratory-gated investigations where the available acquisition time is partitioned across multiple so-called *gates* (typically about 6–10), which substantially increases the noise level in the individual gates compared to that of an ungated acquisition with the same overall duration.

For many years, the only broadly used approach to noise reduction in PET imaging has been the application of low-pass filters — usually Gaussians — which leads to loss of spatial resolution and increased partial volume effects that adversely affect detectability of small lesions as well as quantitative data evaluation.

A way to overcome these problems is the use of locally adaptive edge-preserving filters. One such filter, which exhibits interesting properties and has attracted considerable interest in diverse applications, is the so-called *bilateral filter* (BF) [1]. It acts combinedly in the spatial and in the intensity domain, which allows to reduce image noise while preserving well defined object edges. In the context of PET imaging, the principle usefulness of BF has been previously demonstrated [2, 3]. However, application of this filter requires case-specific tuning of its two free parameters, especially the one acting in the intensity domain. This process can be tedious and might require non-negligible time from the user. The inability to use fixed filter settings and the absence of efficient algorithmic approaches to automatically adjust these filter parameters has made bilateral filtering so far ill-suited for routine clinical use. The present investigation addresses this deficit by proposing to emulate case-specific bilateral filtering with a suitable neural network based approach.

Recently, much work has been done on Deep Learning (DL) based denoisers. In particular, convolutional neural networks (CNN) have been investigated thoroughly in the context of natural and medical image denoising [4]. The special properties of PET imaging, namely a combination of high correlated noise and high dynamic range and the fact that relevant quantitative information is contained in the images that needs to be preserved, makes PET noise reduction a challenging task requiring special care and attention. There are two principally different approaches to DL-based PET denoising [5]: *supervised* and *unsupervised* methods. Supervised DL methods rely on availability of a substantial number of training data sets consisting of pairs of low and high noise PET images. The employed neural networks architectures usually feature various forms of U-Nets [6–10] and generative adversarial networks (GANs) [6, 10, 11]. However, other architectures have been suggested as well [6, 10–15]. As is well known, acquiring a large number of high quality training examples is difficult in medical imaging in general and in PET in particular. Therefore, non-supervised approaches are gaining increased attention. One example is the deep image prior technique [16], which was already successfully applied to static [17] and dynamic [18, 19] PET imaging and has demonstrated improvements over conventional denoising methods. Another family of unsupervised methods relies on training using noisy data only. Examples are the so-called *Noise2Noise* [20], *Noisier2Noise* [21], and *Noise2Void* [22] approaches. However, applied to PET, these methods perform generally worse than commonly used supervised approaches [23, 24], with the modified *Noise2Noise* being the closest contender [25].

Considering the bilateral filter, it has in fact been previously investigated in the context of DL. Specifically, bilateral filters as part of the network design have shown to improve natural image processing tasks [26–28]. Medical imaging applications, however, are so far restricted to denoising of CT and X-Ray data. In this context, bilateral filtering solutions with static learnable kernels [29] and adaptive kernels [30–33] were proposed. However, the possibility of reproducing manually optimized bilateral filtering with a general purpose CNN (e.g. U-Net) has not been investigated before. Moreover, so far no DL-based bilateral filters have been developed explicitly for PET noise reduction in a clinical setting.

In this work, we therefore have investigated to what extent a suitably trained CNN can reproduce the results of manually adjusted case-specific bilateral filtering for single-gate denoising of respiratory-gated PET data with the ultimate aim to use such a filter as an integral preprocessing step in motion correction of such PET investigations. For this purpose, we trained a dedicated U-Net based CNN on a large number of clinical respiratory-gated PET/CT investigations. The training data consisted of pairs of unfiltered and BF processed single-gate images where the filter parameters were optimized separately for each gate. The trained CNN was then applied to separate test data, i.e. image volumes unseen during network training. The resulting CNN-filtered image volumes were quantitatively analysed regarding image noise and signal recovery (*standardized uptake value*, SUV) in suitable three-dimensional regions of interest (ROIs) and compared to the corresponding results obtained in image volumes resulting from individually optimized bilateral filtering of the respective original data set.

## Methods

### Bilateral filter

A bilateral filter operates in two different domains: the filter kernel is defined as the product of a spatial filter $W_S$ and an intensity filter $W_I$ [1]. If both filters have a Gaussian shape and assuming that the three-dimensional spatial filter is isotropic the BF kernel is given by

1 $$\begin{aligned} W(m,n) = \underbrace{\exp {\left[ -\frac{(\textbf{P}_m-\textbf{P}_n)^2}{2\sigma _S^2}\right] }}_{\mathrm{spatial\ domain}\ (W_S)} ~\cdot ~ \underbrace{\exp {\left[ -\frac{(I_m-I_{n})^2}{2\sigma _I^2}\right] }}_{\mathrm{intensity\ domain}\ (W_I)}\, \end{aligned}$$

where *m* is the index of the target voxel at position $\textbf{P}_m$ with intensity $I_m$ and *n* enumerates the neighbouring voxels included in the weighted average computation. $\sigma _S$ and $\sigma _I$ are the free parameters of the filter, representing the standard deviations of the two Gaussian components. In the present study an anisotropic spatial filter was used with reduced width along the axial direction (scale factor: $0.76$) to accommodate for the slightly higher axial resolution of the reconstructed images. In the remainder of this paper, $\sigma _S$ denotes the arithmetic mean $(2 \sigma _{xy} + \sigma _z)/3$ of the actually applied spatial filter. The parameters $\sigma _S$ and $\sigma _I$ determine which distance to the target voxel is considered to be *close* in the respective domain: only voxels that are close to the target voxel *simultaneously* in the spatial as well as the intensity domain will substantially contribute to the value assigned to that target voxel in the resulting, filtered image.

### Patient group and ground truth definition

Altogether, 70 respiratory-gated PET data sets from clinical routine investigations acquired on a Biograph Vision 600 PET/CT system (Siemens Medical Solutions, Knoxville, TN, USA) with different tracers ($[^{18}\text {F}]$FDG: $N = 34$, $[^{18}\text {F}]$L-DOPA: $N = 25$, $[^{68}\text {Ga}]$DOTATATE: $N = 11$) were available for the present investigation. Table 1 provides further information regarding the study cohort. The following clinical vendor-provided gating procedure was used: during PET/CT data acquisition the respiratory signal was recorded using the pressure sensitive belt sensor version of an external respiratory gating system (AZ-733VI; Anzai Medical Co. Ltd., Tokyo, Japan). Using this signal, the PET data was subsequently divided via a manufacturer-specific algorithm resulting in 8 phase-based gates with similar noise characteristics. For each PET gate, the corresponding CT gate was used for attenuation correction. Visual inspection of all data sets led to exclusion of a single $[^{18}\text {F}]$FDG scan with untypical excessively high image noise due to a very low injected dose (132 MBq) and high post-injection scan delay (2.5 h). See Figure S2 in the supplemental material for more details.

**Table 1:** Compilation of patient data, acquisition and image reconstruction details and summary statistics of the performed data analysis

		$[^{18}\text {F}]$FDG	$[^{18}\text {F}]$L-DOPA	$[^{68}\text {Ga}]$DOTATATE	All
**Patient data set**					
N (excluded)		33 (1)	25 (0)	11 (0)	69 (1)
Sex (m/f)		19 / 14	12 / 13	7 / 4	38 / 31
﻿Age	[y]	$\text {69.0}\pm \text {8.3}$	$\text {68.4}\pm \text {9.4}$	${58.5}\pm \text {13.3}$	$\text {67.1}\pm \text {10.2}$
Weight	[kg]	$\text {79.8}\pm \text {17.5}$	$\text {77.4}\pm \text {14.7}$	$\text {83.9}\pm \text {17.7}$	$\text {79.6}\pm \text {16.5}$
Injected dose	[MBq]	$\text {267.3}\pm \text {55.6}$	$\text {229.9}\pm \text {18.8}$	$\text {144.5}\pm \text {19.9}$	
Scan time p.i.	[min]	$\text {98.2}\pm \text {29.8}$	$\text {103.1}\pm \text {27.3}$	$\text {68.0}\pm \text {15.4}$	
Scan duration	[min]	$\text {8.9}\pm \text {3.3}$	$\text {10.1}\pm \text {2.1}$	$\text {6.5}\pm \text {2.2}$	
Respiratory gates		8	8	8	
Recon protocol		PSF+TOF 4i5s	PSF+TOF 4i5s	PSF+TOF 4i5s	
voxel size	[mm^3^]	3.3 × 3.3 × 2.0	3.3 × 3.3 × 2.0	3.3 × 3.3 × 2.0	
Matrix size		220 × 220 × [132–153]	220 × 220 × [132–153]	220 × 220 × [132–153]	
Number of images		264	200	88	552
**BF parameter distribution**					
$\sigma _S$	[mm]	$\text {5.0}\pm \text {0.8}$	$\text {4.9}\pm \text {0.7}$	$\text {4.5}\pm \text {0.7}$	$\text {4.9}\pm \text {0.7}$
$\sigma _I$ (SUV)	[g/mL]	$\text {0.9}\pm \text {0.4}$	$\text {0.8}\pm \text {0.2}$	$\text {4.0}\pm \text {2.3}$	$\text {1.4}\pm \text {1.5}$
**Focal uptake ROIs**					
N (per image)		792 (3)	600 (3)	264 (3)	1656 (3)
$\delta \text {SUV}_\text {max}^{\text {BF},\text {STD}}$	[%]	$\text {-4.8}\pm \text {6.4}$	$\text {-3.4}\pm \text {3.6}$	$\text {-5.5}\pm \text {4.6}$	$\text {-4.4}\pm \text {5.3}$
$\delta \text {SUV}_\text {max}^{\text {CNN},\text {STD}}$	[%]	$\text {-4.3}\pm \text {5.7}$	$\text {-4.1}\pm \text {4.6}$	$\text {-2.5}\pm \text {4.3}$	$\text {-3.9}\pm \text {5.2}$
$\delta \text {SUV}_\text {max}^{\text {CNN},\text {BF}}$	[%]	$\text {0.5}\pm \text {5.8}$	$\text {-0.6}\pm \text {3.2}$	$\text {2.9}\pm \text {3.0}$	$\text {0.5}\pm \text {4.8}$
$\Delta \text {SUV}_\text {max}^{\text {BF},\text {STD}}$	[g/mL]	$\text {-0.33}\pm \text {0.31}$	$\text {-0.26}\pm \text {0.20}$	$\text {-1.63}\pm \text {1.30}$	$\text {-0.51}\pm \text {0.75}$
$\Delta \text {SUV}_\text {max}^{\text {CNN},\text {STD}}$	[g/mL]	$\text {-0.32}\pm \text {0.41}$	$\text {-0.32}\pm \text {0.35}$	$\text {-0.62}\pm \text {1.02}$	$\text {-0.37}\pm \text {0.55}$
$\Delta \text {SUV}_\text {max}^{\text {CNN},\text {BF}}$	[g/mL]	$\text {0.01}\pm \text {0.48}$	$\text {-0.05}\pm \text {0.31}$	$\text {1.01}\pm \text {1.13}$	$\text {0.15}\pm \text {0.70}$
$\text {FLR}^{\text {STD}}$		$\text {12.1}\pm \text {16.3}$	$\text {12.5}\pm \text {13.6}$	$\text {4.5}\pm \text {2.9}$	$\text {11.1}\pm \text {14.3}$
$\text {FLR}^{\text {BF}}$		$\text {12.1}\pm \text {16.4}$	$\text {12.5}\pm \text {13.7}$	$\text {4.4}\pm \text {2.9}$	$\text {11.0}\pm \text {14.4}$
$\text {FLR}^{\text {CNN}}$		$\text {12.0}\pm \text {16.5}$	$\text {12.4}\pm \text {13.8}$	$\text {4.4}\pm \text {2.9}$	$\text {10.9}\pm \text {14.4}$
**Noise level ROIs**					
N (per image)		264 (1)	200 (1)	88 (1)	552 (1)
$\delta \text {Noise}^{\text {BF},\text {STD}}$	[%]	$\text {-85.4}\pm \text {13.3}$	$\text {-88.4}\pm \text {6.1}$	$\text {-71.6}\pm \text {10.4}$	$\text {-84.3}\pm \text {12.1}$
$\delta \text {Noise}^{\text {CNN},\text {STD}}$	[%]	$\text {-80.3}\pm \text {13.2}$	$\text {-85.0}\pm \text {6.5}$	$\text {-65.5}\pm \text {8.3}$	$\text {-79.7}\pm \text {12.4}$
$\delta \text {Noise}^{\text {CNN},\text {BF}}$	[%]	$\text {6.2}\pm \text {12.1}$	$\text {4.2}\pm \text {8.7}$	$\text {7.0}\pm \text {8.3}$	$\text {5.6}\pm \text {10.5}$
$\Delta \text {Noise}^{\text {BF},\text {STD}}$	[pp]	$\text {-12.7}\pm \text {2.9}$	$\text {-13.0}\pm \text {3.3}$	$\text {-9.6}\pm \text {4.3}$	$\text {-12.3}\pm \text {3.5}$
$\Delta \text {Noise}^{\text {CNN},\text {STD}}$	[pp]	$\text {-12.2}\pm \text {2.9}$	$\text {-12.6}\pm \text {3.1}$	$\text {-8.9}\pm \text {3.5}$	$\text {-11.8}\pm \text {3.3}$
$\Delta \text {Noise}^{\text {CNN},\text {BF}}$	[pp]	$\text {0.5}\pm \text {1.1}$	$\text {0.4}\pm \text {0.8}$	$\text {0.7}\pm \text {1.2}$	$\text {0.5}\pm \text {1.0}$

Where appropriate, results are represented as $\text {mean}\pm \text {SD}$

Prior to further data processing, the gated data sets were split into single gates, resulting in a total of 552 single-gate image volumes. For each of these image volumes four three-dimensional ROIs were delineated by two experienced observers using the ROVER software package (version 3.0.73; ABX GmbH, Radeberg, Germany). For image noise assessment, one ROI was placed in a visually homogeneous liver area. The remaining three ROIs in each image volume were positioned on different target structures with elevated focal uptake at different target to background contrast levels. These ROIs mostly correspond to tumor lesions but partly also to areas in the myocardium or excretory system. Wherever possible, the three ROIs were furthermore selected in such a way that their $\text {SUV}_\text {max}$ differed by at least a factor of 1.5–2 in order to achieve adequate coverage of dynamic range.

Due to the large number of 552 image volumes for which case specific user-approved BF filter parameter settings are required, it was deemed necessary to facilitate this process by utilizing a dedicated automated optimization procedure that we implemented specifically for this project. The procedure performs a brute force grid search of the two-dimensional BF parameter space for each patient, minimizing the function

2 $$\begin{aligned} S(\sigma _S,\sigma _I) = \sum _{i=1}^{g}\sum _{j=1}^{l} {\left[ \delta {\text {SUV}_\text {max}^{i,j}(\sigma _S,\sigma _I)}\right] ^2} + \sum _{i=1}^{g} {\left[ \delta {\text {Noise}^{i}(\sigma _S,\sigma _I)}\right] ^2} \end{aligned}$$

where $\delta {\text {SUV}_\text {max}^{i,j}(\sigma _S,\sigma _I)}$ and $\delta {\text {Noise}^{i}(\sigma _S,\sigma _I)}$ are the fractional differences between original (unfiltered) and BF-processed gate-specific $\text {SUV}_\text {max}$ and noise level, respectively, for gate *i*, focal uptake ROI *j*, and filter parameters $\sigma _S$ and $\sigma _I$ (see Eqs. 4 and 5 for definitions of $\delta {\text {SUV}_\text {max}}$ and noise level). Summation extends over the given $g = 8$ gates and $l = 3$ focal “lesion” ROIs per patient. The optimization is performed jointly for all gates per patient to improve stability of the process and with the rationale that all gates within a single gated study exhibit essentially identical image properties (noise, spatial resolution, contrast) so that the same filter settings should be suitable/optimal for all gates. Note that the gate-specific unfiltered $\text {SUV}_\text {max}$ and the noise-level of the ungated data (summed over all gates) serve as a reference in the above equation.

The objective thus can be paraphrased as striving to make the BF-processed single gates match the noise level of the ungated summed image without causing relevant signal loss, thus $\text {SUV}_\text {max}$ reduction, in hot spots.

Optimization was performed by scanning a 11 × 15 grid of $\sigma _S$ and $\sigma _I$ values covering a range from 3.1 to 5.9 mm for $\sigma _S$ and from 0.1 to 10.0 SUV for $\sigma _I$. This process generates 165 bilaterally filtered image volumes for each of the 552 single-gate data sets, thus altogether 91080 individually BF-processed PET image volumes. Given the nature of bilateral filters — locally variant, inability to factor 3D filtering into successive 1D filtering operations as is possible with spatial Gaussians — the described grid search can be computationally intensive and very time consuming. To reduce processing time with the used bilateral filter solution at hand, computations were distributed over a high performance cluster of 12 nodes with 560 CPU cores and 3.8 TiB available memory.

Finally, the — according to the above explained criterion — optimally BF-processed image volumes underwent visual inspection by two experienced observers and filter parameters were adjusted in cases where this was deemed necessary.

### Data preprocessing and neural network training

As described above, input for network training consisted of pairs of original and optimally bilaterally filtered single gate image volumes. All image volumes had a common voxel size of 3.3 × 3.3 × 2.0 mm^3^ and transaxial matrix size of 220 × 220 and extended axially over 132 to 153 slices. Image intensities in each image volume were normalized to the [0, 1] range. Image patches of 32 × 32 × 32 voxels were extracted with a partial overlap of at least 25%. The whole process resulted in a total of 276048 pairs of volumes used for neural network input. Additional run-time data augmentation was applied in the form of random flips in all 3 dimensions and a gamma correction of both input and output, simultaneously, with $\gamma = (1 + |G|)^{\textrm{sign}(G)}$, where *G* is a normally distributed random variable with zero mean and standard deviation of 0.5.

**Fig. 1:**
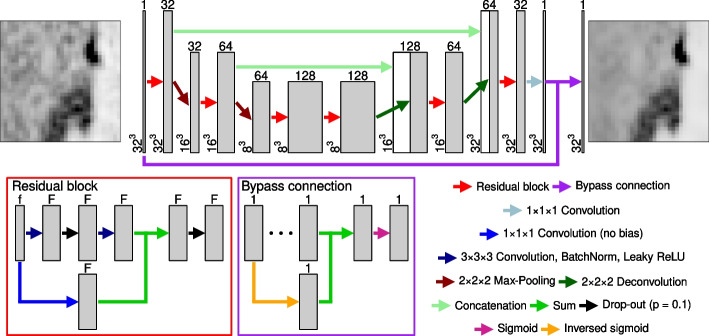
Architecture of the U-Net based CNN. Numbers above and beside each block designate the number of feature channels and matrix size at the given state, respectively. Separate blocks show the structure of residual and bypass connections. Ellipsis in the bypass block designate the main body of the U-Net. The image on the left indicates an example input PET volume and the image on the right is the corresponding output PET volume

For the network, we developed a modified residual 3D U-Net CNN architecture (see Fig. 1). The key modification consists of an additional connection bypassing the whole CNN in a way that only minimal corrections to the input image have to be learned, which corresponds to *residual learning* similar to [6, 8, 34]. In particular, the input image was transformed using an inverse sigmoid (*logit*) function and then added to the output of the U-Net before the final sigmoid activation layer. This way, the output intensities are limited to a [0, 1] range and zero output of the U-Net (i.e. no corrections proposed) translates to an unaltered resulting image. Additionally, the proposed design of the bypass connection makes it difficult for the CNN to change low and high image intensity values, which is beneficial for the task at hand. The Apache MXNet (version 1.9.1) package for the R language and environment for statistical computing (version 4.3.1) [35] was used for implementation. Training was performed on a Ubuntu Linux 22.04.3 system on four NVIDIA V100S Tesla graphic processing units (CUDA: 11.7; driver: 515.65.01) equipped with 32GiB of graphics memory and available 512 GiB of system memory for the two 2.6 GHz Intel Xeon Gold 6132 CPUs, providing a total of 56 processing cores for the preprocessing.

A﻿ 5-fold cross-validation scheme with five equally sized folds was employed to assess the network performance in all data. For each of the five training runs, data from 64% of the patients were assigned for training, 16% for validation, and 20% for testing data sets, respectively while ensuring that all single-gate volumes of selected patient data sets were not split throughout these partitions. Network training was performed for a maximum of 400 epochs with the *Adadelta* optimizer, a batch size of 128 and using a Mean Absolute Error loss function. The training process was monitored by calculating the Mean Squared Error (MSE) metric in the validation phase. Training was stopped if no improvement in the evaluation metric was recorded for 50 epochs. The five models achieving the lowest MSE in the respective validation data were selected for further evaluation.

### Data analysis and data evaluation

Influence of filtering on lesion SUV quantification was assessed by considering the absolute differences

3 $$\begin{aligned} \Delta \text {SUV}_\text {max}^{A,B} = \text {SUV}_\text {max}^A - \text {SUV}_\text {max}^B\, \end{aligned}$$

as well as symmetrical fractional differences

4 $$\begin{aligned} \delta \text {SUV}_\text {max}^{A,B} = \frac{\Delta \text {SUV}_\text {max}^{A,B}}{\left[ \text {SUV}_\text {max}^A+\text {SUV}_\text {max}^B\right] / 2} \times 100\%, \end{aligned}$$

where superscripts *A* and *B* denote the image volume types to be compared (two out of STD: vendor-provided/unfiltered, BF: BF-processed, CNN: CNN-processed). Note, that all possible combinations, i.e. (STD,BF), (STD,CNN), (BF,CNN), were considered.

Furthermore, the image noise level was quantified by

5 $$\begin{aligned} \text {Noise}^A = \frac{\text {SUV}_{\text {sd},\text {liver}}^A}{\text {SUV}_{\text {mean},\text {liver}}^A} \times 100\% \, \end{aligned}$$

where $\text {SUV}_{\text {sd},\text {liver}}^A$ and $\text {SUV}_{\text {mean},\text {liver}}^A$ are the standard deviation and the mean of the SUV values in the liver ROI, respectively. The absolute and fractional noise level differences between image types *A* and *B*, $\Delta \text {Noise}^{A,B}$ and $\delta \text {Noise}^{A,B}$, are defined analogously to the respective $\text {SUV}_\text {max}$ differences.

## Results

**Fig. 2:**
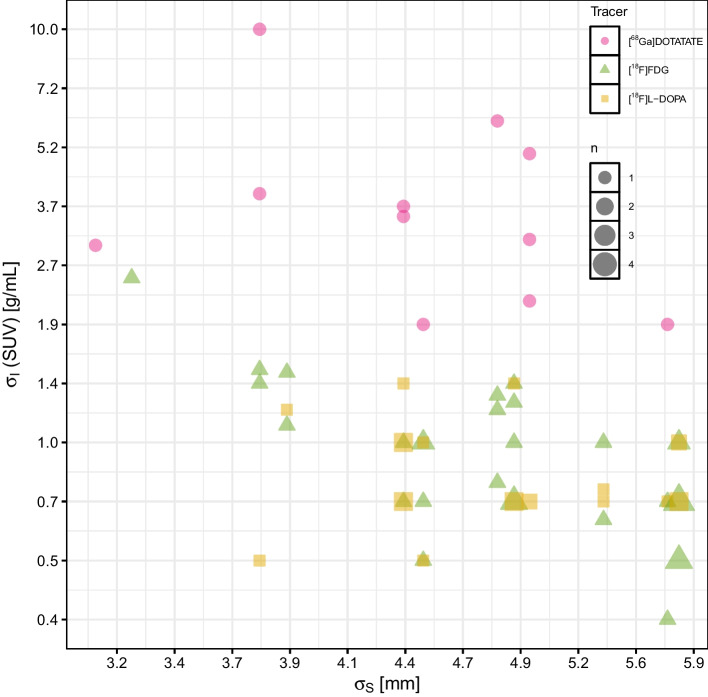
Scatter plot of the bilateral filter parameters $\sigma _I$ and $\sigma _S$ derived for (and applied for filtering of) the 69 gated studies used for neural network training. Note the significant inter-study and inter-tracer variability of the filter parameters

The automated procedure used for optimal filter parameter determination successfully chose adequate filter parameters for 50 out of the 69 patient data sets according to consensus of two experienced observers. The other 19 data sets where automatic filter parameter selection did not produce optimal results were mostly considered to be the result of over-smoothing but partly also exhibiting obvious artifacts such as too abrupt intensity changes at lesion boundaries. For these data sets, the BF parameters where adjusted manually according to the stated objective of maximizing overall noise-reduction while avoiding or minimizing resolution loss in hot lesions without causing too sharp edges. The manual optimization of BF parameter settings took between one and five minutes per patient underlining the desirability of a fully-automated approach for clinical use. After this manual intervention the overall distribution of filter parameter values displayed in Fig. 2 was obtained.

All five neural network training runs finished successfully. The early stop criterion was met in all folds after $286 \pm 53$ epochs. Total training time was approximately 11 days with the aforementioned computational hardware.

**Fig. 3:**
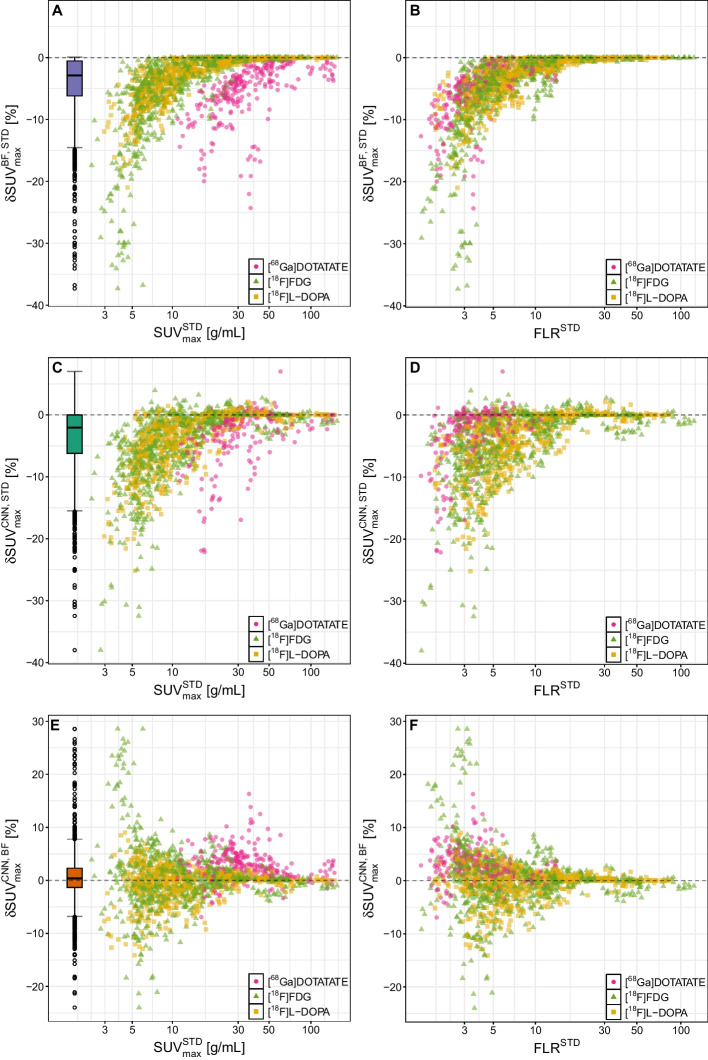
Relative $\delta \text {SUV}_\text {max}$ differences as a function of absolute $\text {SUV}_\text {max}$ (left) and as a function of focal uptake to liver ratio (FLR) (right) in the unprocessed STD images. Top row: BF versus STD, middle row: CNN versus STD, bottom row: CNN versus BF. Note the logarithmic scale of the abscissa

Computation time required for processing a single image volume with the final trained CNN is about 2–4 s on standard computing hardware, which is comparable to the time required for bilateral filtering of the same data. A summary of the quantitative evaluation can be found in Table 1. Evaluation of the focal uptake ROIs revealed that CNN as well as BF processing essentially maintain the focal $\text {SUV}_\text {max}$ values of the unfiltered images with a $\text {mean} \pm \text {SD}$ of $\delta \text {SUV}_\text {max}^{\text {CNN},\text {STD}}=(-3.9\pm 5.2)\%$ and $\delta \text {SUV}_\text {max}^{\text {BF},\text {STD}}= (-4.4\pm 5.3)\%$, respectively. Overall, there is a slight tendency for reduction of $\text {SUV}_\text {max}$ in the CNN and BF images compared to STD, which is to be expected due to the effect of smoothing in the vicinity of the maximum voxel reducing the known noise-induced systematic positive bias exhibited by the $\text {SUV}_\text {max}$ metric. However, in some cases the reduction exceeded 15%, which hints at actual moderate signal recovery loss. Figure 3 illustrates these results. On the left, $\delta \text {SUV}_\text {max}$ is plotted against $\text {SUV}_\text {max}$ determined in the vendor-provided images. The plots on the right show $\delta \text {SUV}_\text {max}$ as a function of the ratio of $\text {SUV}_\text {max}$ and $\text {SUV}_\text {mean}$ in the liver (*focal uptake to liver ratio*, FLR), again determined in the vendor-provided STD images. Results for BF versus STD are shown in the top row and results for CNN versus STD in the middle row. Obviously, $\text {SUV}_\text {max}$ of the ROIs showing large $\delta \text {SUV}_\text {max}$ exhibit a tracer accumulation comparable to the liver uptake.

**Fig. 4:**
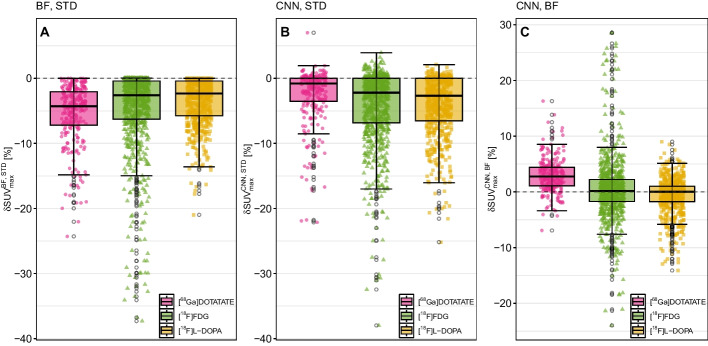
Boxplots of the relative $\delta \text {SUV}_\text {max}$ differences in $\text {SUV}_\text {max}$ separately for the three included tracers **A**: BF versus STD, **B**: CNN versus STD, **C**: CNN versus BF

**Fig. 5:**
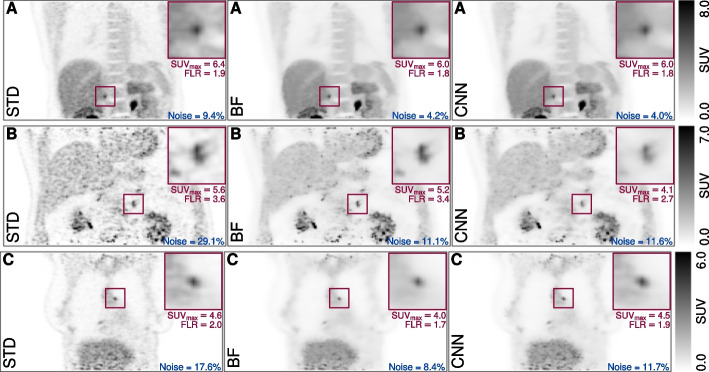
Examples of the differently processed data. Left: vendor-provided/unprocessed; middle: BF processed; right: CNN processed. The top row **A** shows an example ($[^{18}\text {F}]$L-DOPA) of nearly identical performance of BF and CNN. The middle row **B** shows an example ($[^{18}\text {F}]$FDG) with notable reduction of $\text {SUV}_\text {max}$ after CNN processing, which is not present after BF processing. The bottom row **C** shows an example ($[^{18}\text {F}]$FDG) where CNN performs notably better then BF regarding $\text {SUV}_\text {max}$

Regarding the relative performance of CNN and BF, the bottom row in Fig. 3 demonstrates that both methods lead to very similar $\text {SUV}_\text {max}$ values in the vast majority of cases with an overall average difference of $\delta \text {SUV}_\text {max}^{\text {CNN},\text {BF}}=(0.5\pm 4.8)\%$. Still, there are isolated instances with distinctly higher fractional deviations between both methods in the presence of reduced tracer uptake as can be seen in Fig. 3. The boxplots in Fig. 4 show $\delta \text {SUV}_\text {max}$ for the three investigated tracers separately. For $[^{68}\text {Ga}]$DOTATATE there appears to be a small bias towards larger $\text {SUV}_\text {max}$ values after CNN filtering but overall the effect is statistically not significant (*P* = 0.689). Representative examples of BF and CNN processed images are compared to the respective unprocessed images in Fig. 5. Further examples including some corner cases (Figure S1) as well as additional combined violin and boxplots (Figure S3) are provided in the supplementary material.

Evaluation of the noise properties of the differently processed images showed that CNN filtering mostly satisfactorily reproduces the noise level and characteristics of bilateral filtering. $\delta \text {Noise}^{\text {CNN},\text {BF}}$ was on average $(5.6\pm 10.5)\%$. Both, CNN and BF reduced noise compared to their STD image counterparts distinctly, on average by −79.7% and −84.3%, respectively. Overall, noise in the CNN-filtered data was on average only slightly larger than in the BF-filtered images (*P* = 0.006). However, in rare cases $\delta \text {Noise}^{\text {CNN},\text {BF}}$ turned out to exceeded 20%. Visual inspection of the corresponding image data revealed two main reasons for this phenomenon: (a) spurious, noise-induced hotspots, which were removed by BF but not by CNN leading to higher residual noise in the CNN-processed image in comparison to BF, (b) inability to reliably determine the noise metric in the liver due to absence of sufficiently large homogeneous regions within the liver. While (a) is a real effect, (b) rather points to limitations of the available noise metric.

## Discussion

In the present study we have investigated to what extent edge preserving noise reduction in PET, as achieved by case-specific manually tuned (thus, time-consuming) bilateral filtering, can be mimicked by a neural network. Our results clearly show that this is possible. The difference in $\text {SUV}_\text {max}$ between BF and CNN processing was below 10% in the majority of the cases and tracer independent in the considered group of tracers ($[^{18}\text {F}]$FDG, $[^{18}\text {F}]$L-DOPA, $[^{68}\text {Ga}]$DOTATATE), notably including $[^{68}\text {Ga}]$DOTATATE with its extremely high target to background ratios. Nevertheless, in certain situations notable differences between CNN and BF were observed with $\text {SUV}_\text {max}$ differences up to 30%. On closer inspection, all affected ROIs were, however, those with the lowest tracer uptake in the respective image volume and had — intentionally as per the chosen ROI selection strategy — an uptake similar to the liver (see Fig. 3F). The ground truth BF parameters were optimized in such a way as to reduce noise measured in the liver ROI while maintaining $\text {SUV}_\text {max}$ in all other ROIs including the low uptake ROIs. This is challenging when both, focal uptake ROI and liver ROI, have a comparable uptake. Nevertheless, the objective — noise reduction without signal recovery loss — was possible to achieve for most of the low uptake ROIs but not for the above mentioned extreme cases where application of BF leads to a sizable unwanted reduction of $\text {SUV}_\text {max}$ compared to the unfiltered STD data, as can be seen in Fig. 3A, B.

This behaviour might be considered a general limitation of bilateral filtering: even though the filter is locally adaptive, the filter parameters apply globally. They, therefore, cannot be simultaneously chosen in such a way that the filter reduces noise in one region and preserves $\text {SUV}_\text {max}$ in another region when both regions have similar uptake. The root cause is the fact that bilateral filters typically contain a rather broad spatial component characterised by $\sigma _S$, which achieves satisfactory spatial smoothing — and thus noise reduction — in homogeneous regions like the liver. The filter’s intensity component suppresses certain parts of the spatial filter kernel wherever a sizable — as determined by $\sigma _I$ — intensity change (e.g. at object edges) occurs. The filter parameters together thus define, in a certain sense, which intensity changes across the spatial extension of the filter kernel are to be considered as noise and which intensity changes are to be considered as “object edges”. This distinction becomes increasingly less clear-cut when the target’s uptake approaches the general noise level in the image. It thus might be concluded that for very low uptake lesions the bilateral filter starts to act too similar to a purely spatial filter. It is then interesting to note that in our study CNN processing did in fact not over-smooth these low uptake ROIs as severely as BF (see Fig. 3E, F; positive values). On the other hand, CNN processing also exhibited issues in certain corner cases (see Fig. 3C, D) which were better handled by BF. Altogether, it can be stated that both, BF and CNN, can yield unsatisfactory results for target regions with very low uptake. But whenever the uptake clearly exceeds the liver uptake both filters result in comparable $\text {SUV}_\text {max}$ values, with only minor signal recovery loss compared to the original data (see Fig. 3, left column).

Noise after CNN processing was slightly larger than after BF processing with average relative difference of 5.6%. In a few cases, CNN seemingly performed much worse than BF, but closer inspection revealed that this was often a consequence of shortcomings of the noise metric that relies on presence of sufficiently large homogeneous liver regions: in the mentioned cases the liver exhibited unusually heterogeneous tracer distribution and/or multiple lesions, which made determination of the noise metric unreliable.

It should be noted, that the CNN training takes as input only the original data and the BF processed data as the targeted “ground truth”. Information on how the target data are created is not provided. The network does not “know” about the ROIs which were used to optimize the bilateral filter and the network is not trained to reproduce the parameters of the bilateral filter but to reproduce the resulting filtered denoised image. In principle, one might argue, then, that it should have been possible, maybe preferable, to use the low noise sum of all 8 gates as target image in the respective patient instead of generating a target image individually for each gate by application of an optimized BF. In fact, using the summed low noise image would correspond to the usual strategy for training denoisers in PET. Here, too, the usual prerequisite to training of such denoisers is availability of high-quality (low noise) ground truth data. More often than not “high quality” just denotes the typical clinically achievable quality of (ungated) investigations performed with standard protocols regarding scan duration and injected dose. The approach mostly is to just train the network to reproduce that given standard quality when fed with subsets — emulating shortened scan times — of the full data in order to allow dose or scan time reduction in future applications. In the presence of breathing induced motion blurring, however, the training will obviously strive at denoising a motion blurred noisy image volume in such a way as to create a low noise motion blurred image: the denoising cannot be really separated from the general texture properties (including quality of object edge definition, i.e. spatial resolution). Gated images, however, differ in obvious ways from motion blurred images with comparable noise. We thus believe that application of conventionally trained denoisers using ungated training data to gated studies would require further evaluation regarding validity of the results and extent of loss in spatial resolution (as we surmise). On the other hand, using the ungated/summed data in our present study as ground truth instead of the BF-processed single gates would amount to training the network to produce a low noise motion blurred image from a noisy motion-free image. This would obviously be rather pointless in our view.

We therefore believe that — for the special case of respiratory-gated studies — the approach proposed in the present study is offering an interesting, probably superior, alternative to the conventional approach of training the denoiser on subsets of the available full data while using the full data as ground truth. In our view, single-gate denoising will also offer benefits in the context of respiratory motion correction.

Regarding application of DL based denoising in PET, only a few DL studies report on quantitative accuracy of their methods. In [11] noise reduction was performed with a GAN. There, the authors reported a $\text {SUV}_\text {mean}$ bias of $(0.45 \pm 5.59)\%$ in normal tissue and $\text {SUV}_\text {max}$ bias of $(-0.84 \pm 6.94)\%$ in lesions. The same architecture was used in [10] to denoise PET images. Here, a $\text {SUV}_\text {max}$ bias of up to 35% in lung nodules was found. Finally, in [14] a semi-supervised learning approach was used featuring two autoencoders extracting image features at two noise levels with a translation layer in between. For the six evaluated lung nodules $\text {SUV}_\text {max}$ bias was approximately (15–20)%. In our study the $\text {SUV}_\text {max}$ bias was $(0.5\pm 4.8)\%$, which is in line with the result reported in [11]. Also, similar outliers as reported in [10, 14] were present in our investigation.

A limitation of the current study is the above-mentioned partly unreliable determination of the noise level using the liver. Another limitation is the rather low number of included patients (N = 69). Altogether 552 image volumes were included, but each patient contributed 8 respiratory gates representing individual respiratory states. Even though these images are far from being identical, they are of course rather similar, apart from breathing induced relative deformation. This low number of independent datasets could lead to a potentially unbalanced training data set. This is, for example, very likely the cause for the small positive bias of $\text {SUV}_\text {max}$ in CNN filtered $[^{68}\text {Ga}]$DOTATATE data (Fig. 4C). Such data have typically much higher SUV values than measurements with the other two tracers, but were underrepresented in the current data sample (N = 11 versus N = 58). It is also worth noting that the performance of the network was evaluated via the mentioned 5-fold cross-validation scheme only. Further testing with dedicated external data from unseen sources is necessary for better understanding of the CNN performance especially with respect to its generalization capabilities. Thus, inclusion of more data will potentially remove these limitations. Another potential shortcoming is the fact that the bilateral filter was restricted to three spatial dimensions and not extended to 4D by including the temporal dimension (the gates). We believe, however, that it is by no means obvious whether this would have been beneficial since it would potentially require to sacrifice the already low temporal resolution (due to the rather low number of gates) by smoothing along the temporal dimension. Therefore, a separate investigation of the usefulness of 4D bilateral smoothing of gated studies — and, by extension, the corresponding 4D CNNs — would be valuable.

## Conclusions

Our results show that a DL-based denoising utilizing a 3D U-Net CNN architecture can reproduce the results of a case by case optimized BF in a fully automated way. Apart from rare cases, CNN and BF processing lead to images of practically identical quality regarding noise level, edge preservation, and signal recovery. We believe such a network might proof especially useful in the context of improved motion correction of respiratory-gated PET studies but can also help to establish BF-equivalent edge-preserving CNN filtering in clinical PET since it obviates time consuming manual BF parameter tuning.

### Supplementary Information

Supplementary Material 1.﻿

## Data Availability

The datasets generated and/or analyzed during the current study are available from the corresponding author on reasonable request.
