# Supplementary material for: Deep learning based bilateral filtering for edge-preserving denoising of respiratory-gated PET
Source: EJNMMI Phys. 2024 Jul 9;11:58. doi: 10.1186/s40658-024-00661-z (PMC11231129; doi:10.1186/s40658-024-00661-z)
Supplement: Supplementary file 1 — Supplementary Material 1.﻿ [file 40658_2024_661_MOESM1_ESM.pdf]

Supplemental material

Deep Learning based bilateral filtering for edge-preserving denoising of respiratory-gated PET

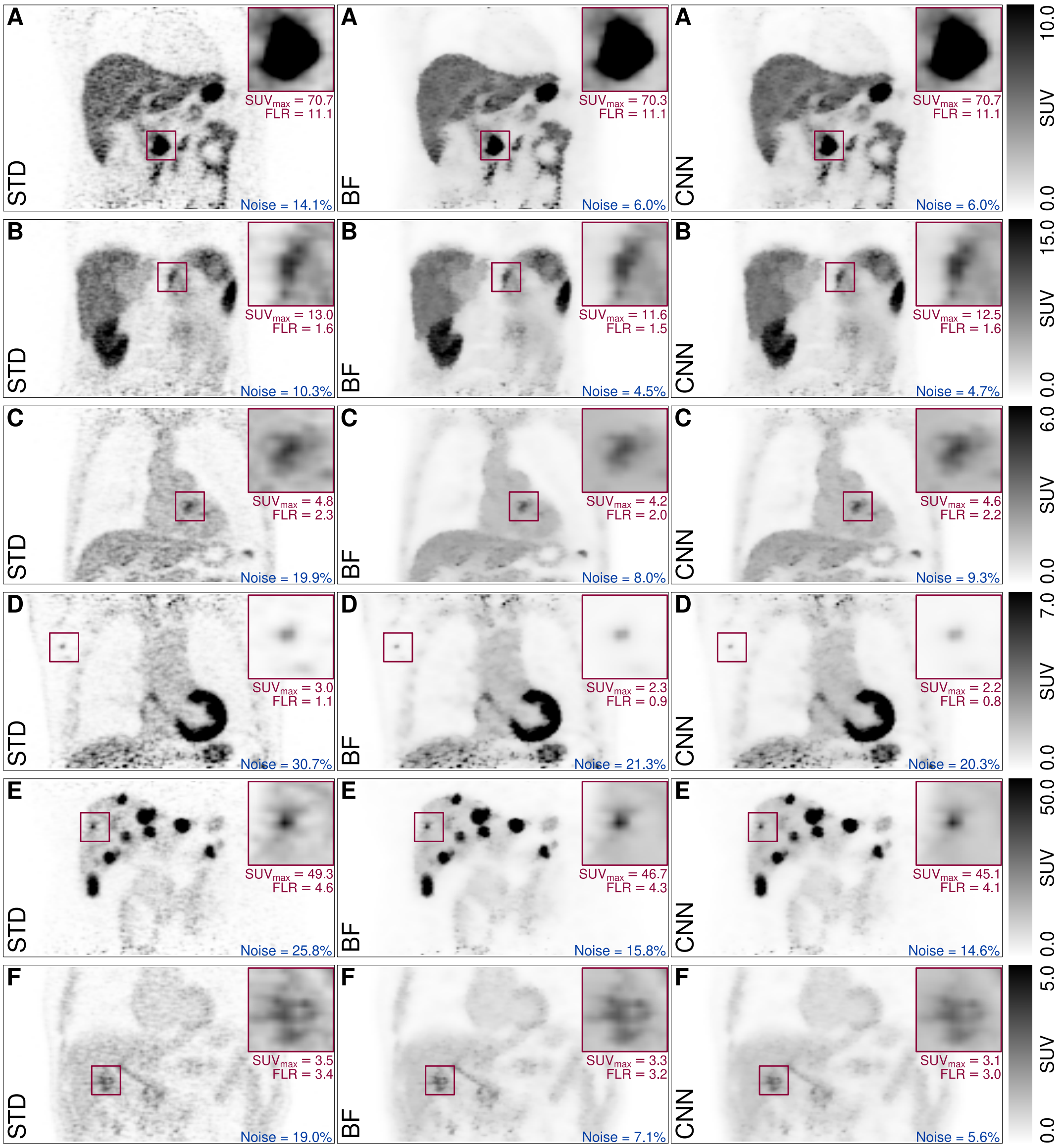

Fig. S1 Examples of the differently processed data. Left: unprocessed, middle BF processed, right CNN processed. **(A)**, [^{68}Ga]DOTATATE: BF and CNN perform almost identical. **(B)**, [^{68}Ga]DOTATATE: BF and CNN perform similar regarding noise reduction but CNN preserves SUV_{max} better. **(C)**, [^{18}F]FDG: slightly inferior noise reduction by CNN but better preservation of SUV_{max}. **(D)**, [^{18}F]FDG: sizable SUV_{max} reduction with both, BF and CNN, for the low uptake lesion (note: noise quantification unreliable since liver not covered by FOV). **(E)**, [^{68}Ga]DOTATATE: essentially identical SUV_{max} preservation with CNN and BF (note: noise quantification unreliable due to absence of sufficiently homogeneous liver region). **(F)**, [^{18}F]L-DOPA: CNN performs better regarding noise reduction but BF preserves SUV_{max}.

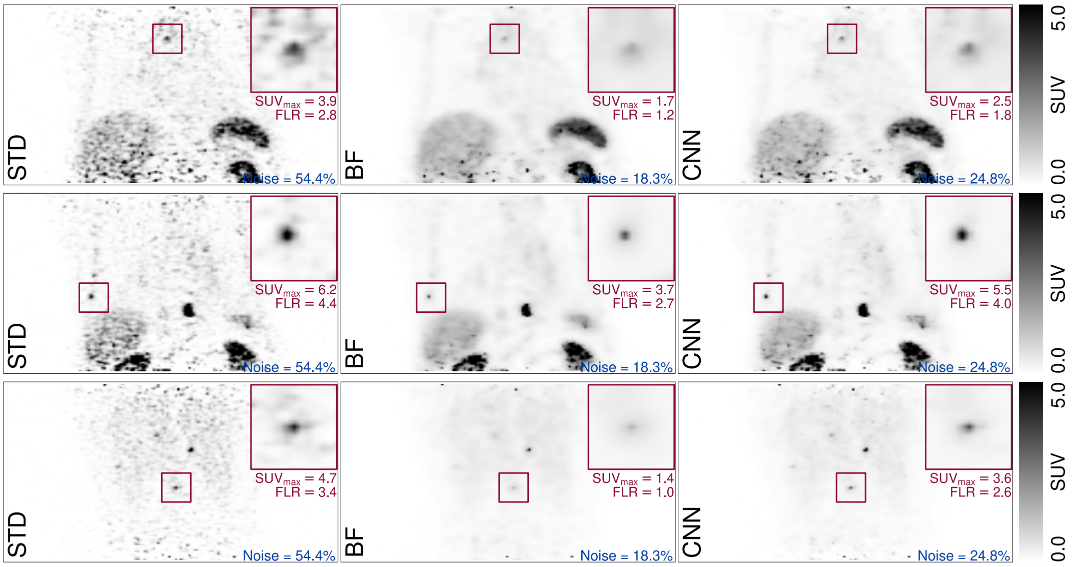

Fig. S2 Image data of the single patient excluded from the dataset due to unusually high noise level (> 50%) in the unprocessed (STD) image due to a low injected dose (132 MBq) and high post-injection scan delay (2.5 h). Left: unprocessed, middle BF processed, right CNN processed (5-fold ensemble prediction). Rows correspond to different coronal slices. Note, that manual optimization of BF according to our predefined criteria was impossible. Presented is BF with automatically selected parameters. Both, BF and CNN are prone to produce spurious focal uptake structures especially at the edges of the field-of-view. Therefore, special care needs to be taken when applying CNN or BF to exceedingly noisy data.

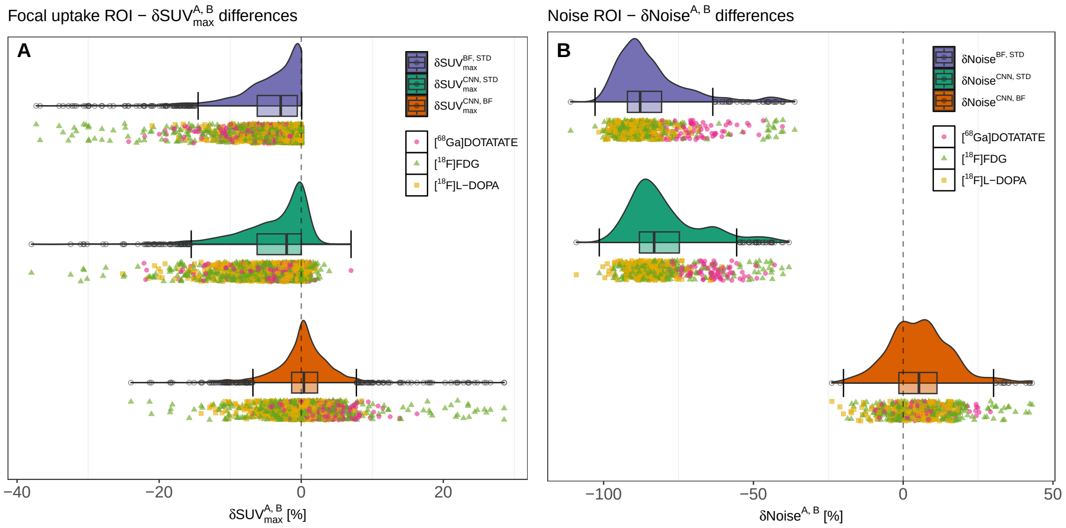

Fig. S3 Combined probability density and boxplots of the distribution of percentage differences $\delta \mathrm{SUV}^{\mathrm{A,B}}_{\max}$ for all focal uptake ROI $\mathrm{SUV}_{\max}$ values (**A**) as well as $\delta \mathrm{Noise}^{\mathrm{A,B}}$ for all noise level ROI values (**B**) for the indicated pairs from CNN, BF, and STD.
